# Functional Activity of Cytokine-Induced Killer Cells Enhanced by CAR-CD19 Modification or by Soluble Bispecific Antibody Blinatumomab

**DOI:** 10.3390/antib13030071

**Published:** 2024-08-30

**Authors:** Silvia Zaninelli, Silvia Panna, Sarah Tettamanti, Giusi Melita, Andrea Doni, Francesca D’Autilia, Rut Valgardsdottir, Elisa Gotti, Alessandro Rambaldi, Josée Golay, Martino Introna

**Affiliations:** 1 Center of Cellular Therapy “G. Lanzani”, Division of Hematology, ASST Papa Giovanni XXIII, 24122 Bergamo, Italy; zaninelli.silvia@gmail.com (S.Z.);; 2 M. Tettamanti Center, Fondazione IRCCS San Gerardo dei Tintori, 20900 Monza, Italy; 3 Unit of Multiscale and Nanostructural Imaging, IRCCS Humanitas Research Hospital, 20089 Milano, Italy; 4 Hematology and Bone Marrow Transplant Unit, ASST Papa Giovanni XXIII Hospital, 24127 Bergamo, Italy; 5 Department of Oncology and Hematology, Università degli Studi di Milano, 20122 Milan, Italy

**Keywords:** chimeric antigen receptor, bispecific antibody, cytokine-induced killer, B-cell neoplasia, CAR signaling

## Abstract

Strategies to increase the anti-tumor efficacy of cytokine-induced killer cells (CIKs) include genetic modification with chimeric antigen receptors (CARs) or the addition of soluble T-cell engaging bispecific antibodies (BsAbs). Here, CIKs were modified using a transposon system integrating two distinct anti-CD19 CARs (CAR-MNZ and CAR-BG2) or combined with soluble CD3xCD19 BsAb blinatumomab (CIK + Blina). CAR-MNZ bearing the CD28-OX40-CD3ζ signaling modules, and CAR-BG2, designed on the Tisagenlecleucel CAR sequence (Kymriah^®^), carrying the 4-1BB and CD3ζ signaling elements, were employed. After transfection and CIK expansion, cells expressed CAR-CD19 to a similar extent (35.9% CAR-MNZ and 17.7% CAR-BG2). In vitro evaluations demonstrated robust proliferation and cytotoxicity (~50% cytotoxicity) of CARCIK-MNZ, CARCIK-BG2, and CIK + Blina against CD19^+^ target cells, suggesting similar efficacy. All effectors formed an increased number of synapses, activated NFAT and NFkB, and secreted IL-2 and IFN-ɣ upon encountering targets. CIK + Blina displayed strongest NFAT and IFN-ɣ induction, whereas CARCIK-BG2 demonstrated superior synapse formation. All the effectors have shown therapeutic activity in vivo against the CD19^+^ Daudi tumor model, with CARCIK cells showing a more durable response compared to CIK + Blina, likely due to the short half-life of Blina in this model.

## 1. Introduction

Cytokine-induced killer cells (CIKs) are CD3^+^CD56^+^ T lymphocytes expanded in vitro that have cytotoxicity and tumor-homing capacity [1,2,3,4,5], without significant graft-versus-host disease (GvHD) [6,7,8,9]. Allogenic CIKs have been used in several clinical trials to treat hematological and solid cancers [10]. However, CIKs alone have shown therapeutic activity mostly in a low-tumor burden context, underscoring the need for strategies aimed at increasing their cytotoxic activity and tumor specificity.

The activity of CIKs can be redirected towards tumor targets by the combination with bispecific antibodies (BsAbs) simultaneously targeting a CIK surface antigen (e.g., CD5 or CD3) along with a tumor antigen, such as the T-cell engager CD3xCD19 blinatumomab [11,12,13,14,15,16,17]. Another approach involves the genetic modification of CIKs to express chimeric antigen receptors (CARs). Several T-cell-based products modified by viral infection with anti-CD19 CARs have been approved for B-cell acute lymphoblastic leukemia (B-ALL) and B-cell non-Hodgkin lymphoma (B-NHL) treatment [18,19,20,21,22,23]. However, manipulation of viral vectors requires high containment levels and extensive quality controls for safety. Moreover, patient-derived T-cells can fail to expand in vitro when derived from heavily pre-treated patients. To overcome these hurdles, another approach, proposed by our collaborators in Monza [24,25], employs allogenic CIKs as effectors and the Sleeping Beauty (SB) transposon system for stable non-viral cell modification. The phase I/IIa clinical trials are demonstrating that CARCIK-CD19 cells (utilizing a third generation anti-CD19 CAR equipped with CD28 and OX40 domains) can expand and persist in vivo in B-ALL patients and achieve anti-leukemic activity without severe toxicities (FT01CARCIK and FT03CARCIK; Eudract n. 2017-000900-38 and 2020-005025-85) [26].

To better define the mechanism of action and relative efficacies of the different approaches available to enhance CIK cell activity, our study aimed at evaluating and comparing the in vitro and in vivo functional activities of CIKs, either combined with soluble CD3xCD19 bispecific antibody blinatumomab (CIK + Blina) or modified with two different anti-CD19 CAR molecules carrying different signaling modules but sharing the same anti-CD19 moiety. Specifically, CAR-MNZ is the same anti-CD19 CAR used by our groups in clinical trials [24], whereas CAR-BG2 recapitulates the Tisagenlecleucel product (Novartis) [27], albeit cloned in a transposon vector.

## 2. Materials and Methods

### 2.1. Cell Lines and Primary Cells

The B-cell lines REH and Daudi, and T-cell lines Jurkat and HuT 78 were maintained in culture in RPMI1640 medium (Euroclone, Wetherby, West Yorkshire, UK) supplemented with 10% heat-inactivated Fetal Bovine Serum (FBS) (Euroclone), 2 mM L-Glutamine (Euroclone), and 100 μM gentamycin (PHT Pharma, Milano, Italy). 

Peripheral blood mononuclear cells (PBMCs) from normal donors’ buffy coats were obtained by Ficoll Hypaque (Cedarlane, Burlington, Canada) gradient centrifugation. Written informed consent was obtained from all participants prior to sample collection and the study protocol was approved from the ethical committee of Bergamo, Hospital ASST Papa Giovanni XXIII (Project: “Development of novel strategies to redirect immune cells towards tumors, using bispecific antibodies or CAR”, approved on 13 November 2018).

### 2.2. Transposon Plasmids

The anti-CD19 CAR-MNZ transposon plasmid expresses the human third generation anti-CD19-CD28-OX40-CD3ζ CAR under the pTMNDU3 promoter. The CAR coding sequence is flanked by the recognition sites for the Sleeping Beauty (SB) transposase SB11 [24]. The anti-CD19 CAR-BG2 transposon plasmid expresses the human second generation anti-CD19-4-1BB-CD3ζ CAR, based on the published sequence [27], under the EF1α promoter. This sequence was synthetized by GeneArt (Thermo Fisher Scientific, Waltham, MA, USA) and subcloned into the pT4 vector, in which the CAR coding sequence is flanked by the recognition sites for the more efficient SB transposase SB100X. The SB100X transposase plasmid pCMV(CAT)T7-SB100 was kindly provided by Zsuzsanna Izsvak (Addgene #34879; Watertown, MA, USA) [28].

### 2.3. Generation of Unmodified Cytokine-Induced Killer (CIK) and CARCIK-CD19 Cells 

CIK and CARCIK-CD19 were generated starting from PBMCs as previously described [3,24]. Briefly, in both cases, cells were stimulated on day 0 with 1000 U/mL IFN-γ (Clinigen Healthcare Ltd., Burton upon Trent, UK) and on day 1 with 50 ng/mL anti-CD3 (OKT-3, TakaraBio, Kyoto, Japan) and 300 U/mL rhIL-2 (Proleukin, Clinigen Healthcare Ltd.), the latter being included in the medium from day 1 onwards. Expansion was performed for 21 days. For CARCIK-CD19, 10 × 10^6^ PBMCs were first transfected at day 0 with 10–15 μg CAR plasmid and 1 or 5 μg SB11 or SB100X transposase plasmid, respectively, using the human T-cell nucleofector kit (Lonza, Basel, Switzerland) and the Amaxa IIb nucleofector device (Lonza) and placed in culture as indicated above but with the addition of 1.5 × 10^6^ irradiated PBMCs [24]. For in vitro functional assays, ten days after transfection the anti-CD19 CAR^+^ cells were purified by labeling with the poly-histidine tagged recombinant human CD19 protein (His-rhCD19, Acro biosystems, Newark, DE, USA), followed by the anti-histidine FITC antibody and immunoselected through an anti-FITC magnetic beads separation column (Miltenyi Biotec, Bergisch, Gladbach, Germany). The positive fraction was collected and maintained in culture until day 21. 

### 2.4. Flow Cytometry

Immunophenotyping of CIKs and CARCIK-CD19 was performed by staining with anti-CD3-PerCP-Cy5.5 (SK7 clone), anti-CD56-BV510 (NCAM16.2 clone), anti-CD4-PE-Cy7 (SK3 clone), anti-CD8-APC-H7 (SK1 clone), anti-CD45RA-FITC (L48 clone), anti-CD62L-APC (SK11 clone) (all from BD biosciences, San José, CA, USA). CAR detection was achieved by staining with His-rhCD19 and then FITC, APC or PE-conjugated anti-histidine antibody (Miltenyi Biotec) [29]. A FACScanto II flow cytometer device (BD Biosciences) and BD FACSDiva Software version 8.0 were used for analysis.

### 2.5. Cytotoxicity

Cell lysis was evaluated using the calcein release assay as previously described [30]. 

### 2.6. Proliferation

For proliferation assays, CIK or CARCIK-CD19, collected at the end of culture, were labeled with 1 μM 5(6)-Carboxyfluorescein diacetate *N*-succinimidyl ester (CFSE, Sigma-Aldrich, Milan, Italy) and then plated with target cells at different E:T ratios. In the case of CIKs, 10 ng/mL blinatumomab was also added. After 4 days, cells were collected and CFSE expression was analyzed by flow cytometry, using the ModFit LT^TM^ software (version 3.2) to calculate the proliferation index.

### 2.7. Intracellular Cytokines

To measure cytokine production, effector and target cells were co-cultured for six hours at a 1:1 E:T ratio in presence of BD GolgiStop solution (BD Bioscience). Cells were then collected, fixed, and permeabilized using the BD Cytofix/Cytoperm kit (BD Bioscience) following the manufacturer’s instructions and stained with CD3-PerCP-Cy5.5, CD4-PE-Cy7, CD8-APC-H7 (BD Bioscience), IFN-γ-FITC, and IL-2-PE antibodies (Miltenyi Biotec) for flow cytometry analysis.

### 2.8. NF-kB and NFAT Signaling

The 1 × 10^6^ HuT 78 cells were co-transfected with CAR-MNZ or CAR-BG2 and corresponding transposase plasmids. After 7–10 days of expansion, stably transfected CAR^+^ cells were purified by immunoselection. To assess NF-kB and NFAT signaling, 2.5 μg NFAT or NF-kB inducible secreted luciferase reporter plasmids (pNifty3-T-Lucia and pNifty3-N-Lucia, respectively) (InvivoGen, San Diego, CA, USA) were transfected into CAR^+^ or wild type HuT 78, using the nucleofector kit V and Amaxa IIb nucleofector device (Lonza). Twenty-four hours after transfection, cells were counted and co-cultured with the CD19^+^ REH target cell line at 1:1 E:T ratio. Then, 10 ng/mL blinatumomab was added to the unmodified cell lines. After additional 24 h, the light signal was quantified as relative light units (RLUs) on the supernatant incubated with the coelenterazine substrate, using a luminometer (FLUOstar OPTIMA, BMG LABTECH, Cary, NC, USA).

### 2.9. Imaging of Immunological Synapse

The immunological synapse was evaluated by time-lapse microscopy, as detailed in the Supplementary Materials.

### 2.10. In Vivo Animal Model

This study protocol was approved by the Italian Ministry of Health (authorization 768/2021-PR, approval date 27 September 2021). Male NSG mice were sublethally irradiated (200 cGy), and then intravenously (i.v.) injected with the CD19^+^ Burkitt lymphoma cells Daudi. After 1 day, mice were randomized based on the body weight in groups of 5 mice for each condition and i.v. injected with 10 × 10^6^ CARCIK-CD19 cells or an equivalent total number of unmodified CIK. In the latter case, 100 ng blinatumomab was also inoculated i.v. together with CIKs and once a day from Monday to Friday, for the first three weeks after cell infusion. Mice were euthanized, in the absence of the disease, at a pre-defined endpoint, around two times the survival of the untreated mice, to have the possibility to analyze CD3^+^ cells persistence.

### 2.11. Statistical Analyses

Results were compared using the Student’s *t*-test. The Kaplan–Meier method was applied to estimate survival curves, while the log-rank test was used for comparisons. Analyses were performed using GraphPad Prism (v.9.1.1, La Jolla, CA, USA) software. The *p*-values are denoted with asterisks as follows: *p*-value > 0.05: not significant (ns); *: *p* < 0.05; **: *p* < 0.01; ***: *p* < 0.001.

## 3. Results

### 3.1. Characterization of Cytokine-Induced Killer (CIK) and CARCIK-CD19 Cells

**Figure 1:**
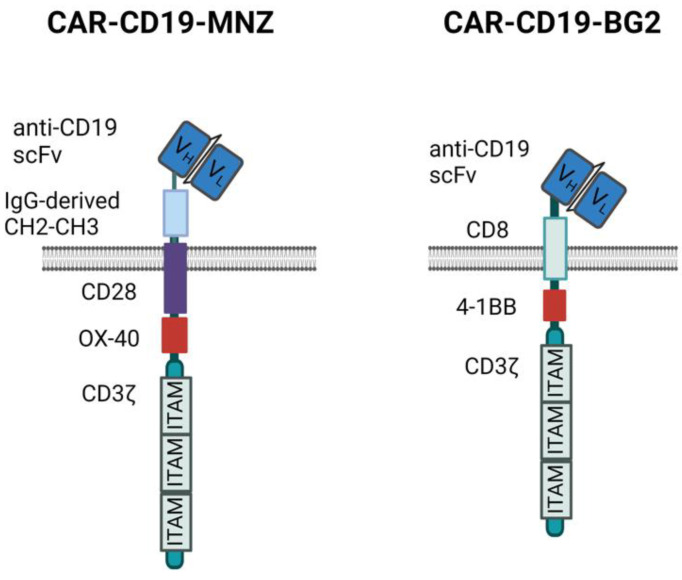
CAR structures. Schematic representation of the two anti-CD19 CAR structures used. Images created with Biorender.com.

We generated two different anti-CD19 CARs carrying the same anti-CD19 scFv sequence but different spacers, transmembrane and signaling domains (Figure 1). 

**Figure 2:**
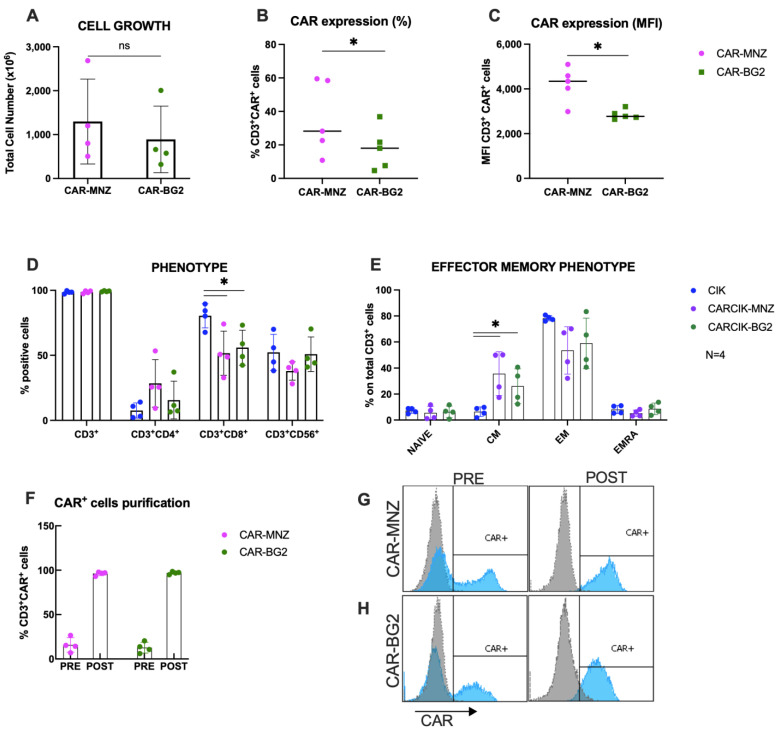
Characterization of CARCIK-CD19 cells. (**A**) PBMCs were transfected with the CAR-MNZ and CAR-BG2 plasmids and expanded to CIK for 21 days. Total cell number at the end of the culture is shown, starting from the same number of cells in both cases (10 × 10^6^ cells). (**B**,**C**) Percentage (**B**) and MFI (**C**) of CAR expression on CD3^+^ cells at the end of culture. (**D**,**E**) Immunophenotype was analyzed at the end of the cultures (day 21) by flow cytometry, including percentages of CD3^+^, CD3^+^CD4^+^, CD3^+^CD8^+^, CD3^+^CD56^+^ (**D**) and effector memory populations (**E**); (**F**–**H**) CAR^+^ cells were purified at day 10–14 by immunoselection. Percentages of CAR^+^ cells pre- and post-purification are shown. Representative flow cytometry histograms of CAR expression of non-purified and purified CARCIK-CD19 (respectively, CAR-MNZ 26.6%, 4588 MFI, and 97.1%, 4031 MFI; CAR-BG2 20.4%, 2641 MFI, and 96.8%, 3208 MFI). The results are the means and standard deviations of four to five experiments using different donors as starting material. (*: *p* < 0.05, ns: not significant).

Following nucleofection with SB transposase plasmids, CARCIK-MNZ and CARCIK-BG2 cells were generated, yielding mean total nucleated cell counts (TNCs) of 1300 × 10^6^ and 891 × 10^6^, respectively, after 21 days expansion starting from 10 × 10^6^ PBMC (Figure 2A). CAR expression levels in the final cell products were observed to be 35.9% and 17.7% for CARCIK-MNZ and CARCIK-BG2 (Figure 2B), with Mean Fluorescent Intensities (MFIs) of 4209 and 2850, respectively (Figure 2C). CARCIK-BG2 exhibited significantly lower CAR expression levels compared to CARCIK-MNZ, both in terms of percentage and MFI (*p* < 0.05).

Phenotypic characterization of the products showed that the percentage of CD3^+^CD56^+^ double-positive CIKs was comparable between transfected and unmodified CIK cultures. The CD8^+^ subpopulation was over-represented in unmodified CIKs (80.4% on total CD3^+^ cells) compared to CARCIK-MNZ and CARCIK-BG2 cultures (respectively 51.6% and 55.9%) (*p* < 0.05; Figure 2D). The effector memory phenotype was comparable between CIK, CARCIK-MNZ and CARCIK-BG2 in terms of naïve, effector memory (EM) and EMRA subpopulations. Instead, central memory (CM) cells were significantly more abundant in CARCIK-CD19 products (35.7% for CARCIK-MNZ and 26.1% for CARCIK-BG2, compared to 6.5% for unmodified CIK cells) (*p* < 0.05; Figure 2E). These phenotypes are intended on total CD3^+^ cells (to compare unmodified CIK with CARCIK); however, the phenotype of CAR^+^ and CAR^−^ populations was also measured and found to be comparable to that obtained on total CD3^+^. Since CAR-CD19 was expressed at slightly but significantly different levels in CAR-MNZ and CAR-BG2 cultures, for further comparative experiments in vitro, we routinely purified CAR^+^ cells at day 10–12 of culture and then further expanded them to obtain comparable cell products (>90% of CAR-CD19) (Figure 2F–H).

### 3.2. Cytokine-Induced Killer (CIK) In Vitro Functional Activity Is Enhanced in Presence of Blinatumomab or Anti-CD19 Chimeric Antigen Receptors (CARs)

**Figure 3:**
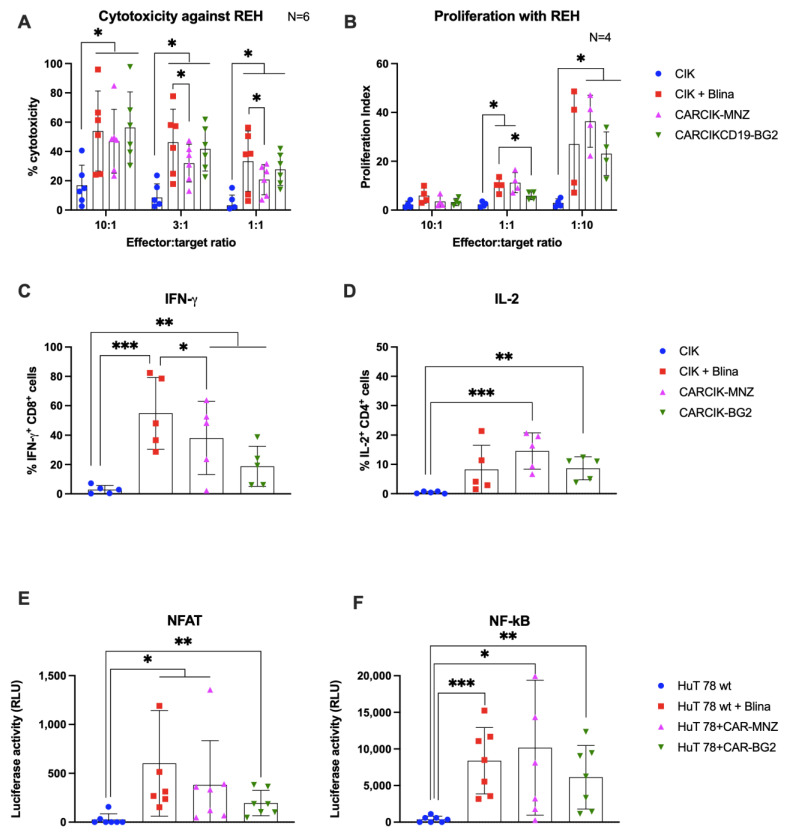
In vitro activity. (**A**) Comparison of the killing activity in vitro using the calcein release assay. CIK (blue bars), CIK in presence of 10 ng/mL blinatumomab (red bars), CARCIK-MNZ (pink bars), and CARCIK-BG2 (green bars) at the end of culture were used as cytotoxic effector cells against the REH cell line at a 10:1, 3:1, and 1:1 E:T ratio. Percentage target lysis is shown as mean and standard deviation of six experiments. (**B**) Comparison of the proliferation ability in vitro (proliferation index) measured by flow cytometry on CFSE-stained effector cells, after co-culture with the target cell line REH, at a 10:1, 1:1, and 1:10 E:T ratio, for 4 days. Proliferation indexes are shown as mean and standard deviation of four experiments. (**C**,**D**) IFN-γ and IL-2 production was determined by intracytoplasmic flow cytometry after 6 h co-culture at 1:1 E:T ratio with REH cell line. Percentages of positive cells are shown as mean and standard deviation of five experiments. (**E**,**F**) The NFAT and NF-kB upon stimulation was measured by co-transfection of Lucia luciferase reporter plasmids into HuT 78 cell line wild type alone (as CIK, blue bars), or stimulated with 10 ng/mL blinatumomab (red bars), or stably transfected with CAR-MNZ (pink bars) or with CAR-BG2 (green bars) and co-cultured with REH cell line for 24 h. Relative luminescence units (RLUs) are shown as mean and standard deviation of five experiments. Columns represent the mean and bars the standard deviation (* *p* < 0.05, ** *p* < 0.01, *** *p* < 0.001).

Purified CARCIK-CD19 or unmodified CIK with the addition of blinatumomab were then evaluated for their ability to mount an in vitro anti-leukemia cytotoxic response against two CD19^+^ cell lines, the B-ALL REH and the Burkitt lymphoma Daudi. The cytotoxic activity of CIK + Blina, along with the two different CARCIK-CD19 products, were significantly higher against both targets (up to 58% and 70%) compared to CIKs alone (18% and 35, respectively), with higher efficacy observed at higher E:T ratios, as expected (Figure 3A and Supplementary Figure S1A). Notably, cytotoxicity induced by CIK + Blina in vitro tended to be slightly higher than that of CARCIK-CD19, and this was statistically significant at the lower suboptimal E:T ratios (Figure 3A and Supplementary Figure S1A).

The addition of Blina or CAR-CD19 modification enhanced the proliferation of effector cells in response to the target line REH, as compared to unmodified CIK (Figure 3B). Proliferation was higher at a low E:T ratio (1:10) than at a high E:T ratio (10:1), indicating that CIKs exhibited significantly higher proliferation in presence of higher amount of target antigen (at 1:10 E:T ratio, *p* < 0.05). No significant difference in proliferation was observed between CIK + Blina and CARCIK-CD19.

We next investigated the production of IFN-γ by CD8^+^ cells and IL-2 by CD4^+^ cells, in response to REH or Daudi target cells. In all cases, the presence of CARs or Blina led to increased levels of IFN-ɣ and IL-2. CIK + Blina produced significantly more IFN-γ than CARCIK-CD19 (*p* < 0.05, Figure 3C and Supplementary Figure S1B). CARCIK-MNZ produced overall more IL-2 than CIK + Blina or CARCIK-BG2, although this was statistically significant only with the Daudi cell line as target (*p* < 0.05, Figure 3D and Supplementary Figure S1C).

The relative activation of the NFAT and NF-kB transcription factors was next investigated using the CD3^+^ HuT 78 cell line, either wild type or stably transfected with the two different CARs. Blinatumomab or the CARs induced significant levels of both NFAT and NF-kB in presence of REH target (*p* < 0.05), with a trend for higher NFAT activation by CIK + Blina and CAR-MNZ (Figure 3E,F).

### 3.3. Chimeric Antigen Receptors (CARs) and Blina Enhance the Immunological Synapse between Cytokine-Induced Killer (CIK) and Target Cells

**Figure 4:**
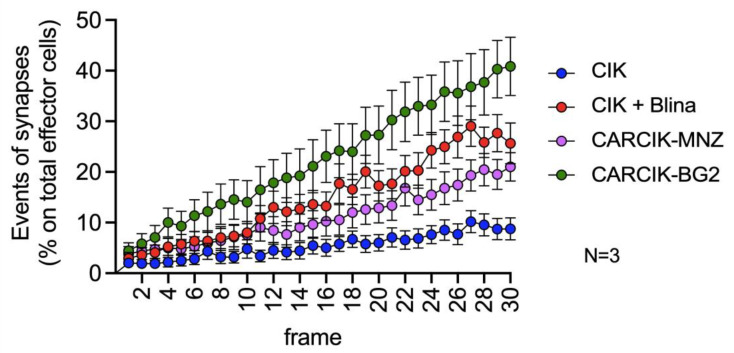
Immunological synapse. Synapses events. All conditions are significatively different from each other (*p* < 0.01). Results are mean ± SD of three independent experiments performed.

The critical role of immune synapse formation in determining both the efficacy and potential toxicity of CAR-modified cells and BiTEs is now widely acknowledged and established [31,32,33]. We therefore sought to evaluate the kinetic of immunological synapse formation between CIK + Blina or CARCIK-CD19 cells and REH target cells by time lapse microscopy. CIKs alone exhibited a very low occurrence of immunological synapses, normalized on the total number of effector cells present in the field of observation (Figure 4 and Supplementary Videos). However, the presence of blinatumomab or CAR-CD19 led to a significant increase in immunological synapse formation over time. Among these, CARCIK-BG2 generated the highest number of synapses, while blinatumomab and CARCIK-MNZ induced a slightly lower frequency of synaptic events (Figure 4).

### 3.4. Blina and Chimeric Antigen Receptors (CARs) Improve Cytokine-Induced Killer Cells (CIKs) In Vivo Anti-Tumor Efficacy 

**Figure 5:**
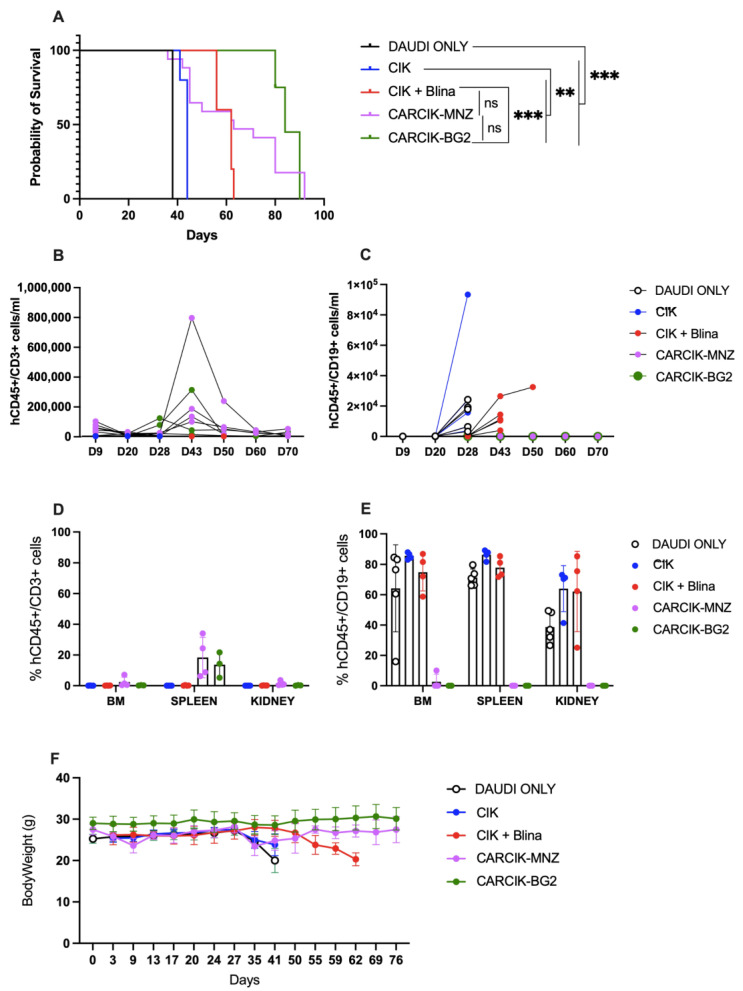
In vivo activity. (**A**) Kaplan–Meier survival curves of NSG mice engrafted with Daudi cells, left untreated (DAUDI only), or treated with CIKs, alone or receiving blinatumomab for the first three weeks, CARCIK-MNZ and CARCIK-BG2. (**B**) Analysis of hCD45^+^CD3^+^ cells/mL in the PB of animals treated with the different effectors. The data with DAUDI only condition (<63 hCD45^+^CD19^+^/mL) are not inserted in the graph for major clarity. (**C**) Analysis of hCD45^+^CD19^+^ cells/mL in the PB of animals treated with the different effectors or left untreated. (**D**) Percentages of hCD45^+^CD3^+^ cells in the BM, spleen, and kidney. (**E**) Percentages of hCD45^+^CD19^+^ cells in the BM, spleen, and kidney. (**F**) Mice body weight after the treatment. (** *p* < 0.01, *** *p* < 0.001, ns: not significant).

Finally, the ability of CIKs in presence of blinatumomab and CARCIK-CD19 to improve survival in vivo was evaluated in a previously established Daudi xenograft model in NSG mice [25]. CIKs alone had limited activity in this model, with a median survival time (MST) of 44 days, compared to 38 days for untreated mice. In contrast, the infusion of blinatumomab for the first three weeks of CIKs treatment significantly improved mice survival with an MST of 62 days (*p* < 0.01 compared to untreated controls or to CIKs). Notably, both CARCIK-MNZ and CARCIK-BG2 induced an even more remarkable enhancement of survival as compared to CIK + Blina, with a median overall survival of 63 and 84 days, respectively (*p* < 0.01 compared to both untreated controls and CIKs alone) (Figure 5A). There was no statistically significant difference observed between the two CAR constructs.

Analysis of circulating CD19^+^ tumor cells in the periphery showed that while Blinatumomab delayed tumor growth, it was unable to fully control the disease, with CD19^+^ cells already detectable in peripheral blood (PB) from day 48 (Figure 5C). Conversely, at sacrifice, no or very few CD19^+^ cells were detectable in PB, bone marrow (BM), spleen, and kidneys of mice treated with CARCIK-CD19 (Figure 5C–E). Moreover, CARCIKs exhibited substantial expansion in the PB, peaking between days 28 and 43 up to ~8 × 10^5^ cells/mL (Figure 5B). CD45^+^CD3^+^ cells were still detectable in the spleen at sacrifice (Figure 5D). As expected, the number of human CD45^+^CD3^+^ cells in the PB of control animals left untreated (DAUDI only) was in all cases below a threshold value of 63/mL. Overall, all the three treatment strategies were well tolerated, as demonstrated by the absence of mice body weight loss after treatment (Figure 5F). Mice started to lose weight only when the expansion of CD19^+^ tumor cells occurred due to insufficient disease control in some conditions.

## 4. Discussion

In this study, we compared three approaches to improve the anti-tumor efficacy and specificity of CIK cells against CD19^+^ neoplastic targets: the addition of blinatumomab to unmodified CIKs or genetically modifying CIKs using transposons with two different anti-CD19 CARs carrying different spacer and costimulatory modules, CAR-MNZ (CH2-CH3 spacer and CD28-OX40) [24] and CAR-BG2 (CD8 spacer and 4-1BB) [27].

Transfection of CARs was successful in both cases and led to diminished CD8 population compared to unmodified CIK, together with an increased percentage of the CM subset. These differences are probably due to the basal signaling produced by CARs, even in the absence of target cells [34,35]. Increased CM population could potentially offer an advantage in vivo, as this has been reported to be accompanied by longer persistence of the effector cells [36].

Both CARs and blinatumomab were very effective in boosting the proliferation and cytotoxic activity in vitro of CIKs against CD19^+^ targets, consistent with previous findings with blinatumomab or CARs from our and other groups [14,25]. Notably, the cytolytic activity of CIK + Blina was generally superior to that of CARCIKs, particularly at low suboptimal E:T ratios, potentially due to the higher percentage of CD8^+^ cells in CIKs compared to CARCIK populations. 

All conditions increased signaling events, in particular NFAT and NF-kB activation, as well as IFN-ɣ and IL-2 production, compared to unmodified CIKs. CIK + Blina induced a trend for higher IFN-ɣ production and NFAT and NF-kB activation compared to the CARCIK-CD19 effectors. The strong induction of IFN-γ signaling by blinatumomab has also been demonstrated by other groups [37]. Higher IFN-ɣ may correlate with the higher cytotoxicity observed. Lastly, the kinetic analysis of immune synapse formation showed that CARCIK-BG2 cells induced the highest number of synapses. Interestingly, CIK + Blina, despite showing in vitro higher cytotoxic activity, did not induce a particularly high percentage of synapses. 

The different responses observed in vitro may be the results of the activation of the different signaling modules, which induce overlapping but not identical intracellular pathways [38,39]. CIK + Blina activation is triggered through the natural TCR and, in particular, CD3ε, while CARs signal via CD3ζ and the co-stimulatory modules (Figure 1) [40,41]. Indeed CAR-MNZ, through CD28, activates both NFAT and NF-kB transcription factors, together with OX-40, which also induces the activation of NF-kB. The combination of these two costimulatory domains was demonstrated to increase the release of IL-2 10-fold compared to the CD28 signal alone [42,43,44]. This is consistent with our results, in which CARCIK-MNZ released the highest levels of IL-2 compared to all the effectors and activated more NFAT and NF-kB compared to CAR-BG2. CD28 also recruits Filamin A at the immune synapse site, inducing cytoskeleton reorganization and an enhancement of the strength of the immune synapse [45,46]. On the other hand, CAR-BG2, upon antigen binding, activates the co-stimulus 4-1BB and, as expected, had the lowest induction of NFAT, since 4-1BB induces only NF-kB activation. The signaling cascade associated to 4-1BB correlates with the lower level of cytokines released compared to CAR-MNZ, whereas the higher number of immune synapse formed can be related to a different cytoskeleton reorganization [44,47,48]. The different kinetics of immune synapse formation can be correlated to the different spacers and costimulatory domains, and it has already been demonstrated that even small changes in the CAR construct can enhance the immune synapse formation [49,50]. Notably, the different frequencies of immune synapses were consistent with the different survival observed in vivo.

The efficiency of the three strategies was compared in vivo in the Daudi xenograft model. The two anti-CD19 CARs showed a comparable median survival and a similar expansion in vivo, suggesting that both CAR-CD19 constructs were effective, despite differences in signaling and cytokine induction. While CIK + Blina showed significantly improved therapeutic activity compared to CIK alone, it resulted in a more transient disease control than CARCIK cells. This lower activity can however be attributed to the blinatumomab’s short half-life in vivo (less than 2 h) [51], and intermittent administration only once a day, 5 days a week, for the first three weeks after CIK infusion. The results obtained with CIK + Blina are consistent with our previous reports, but may not fully mimic the results that could be obtained using a similar strategy in clinical settings [14,52], where blinatumomab is usually administrated by continuous infusion. The CIK + BiTE strategy may be an advantage over genetically modified CIK, because it does not require genetic modification and allows to rapidly stop the treatment if any life-threatening side effects were to take place. Moreover, this platform is amenable to be used with other T-cells redirecting BsAbs, against either leukemic targets or solid tumors. Interestingly, recent strategies propose the genetic modification of T-cells to secrete a BiTE, which therefore can be released and act in the proximity of effector cells [53,54]. 

The functional differences observed between CIK + Blina and CARCIK-CD19 can be explained by the different signals activated through antigen binding. Whereas blinatumomab activates CIK cells through CD3ε, the activation through the CARs is via CD3ζ. In addition to the contribution of CD3 ITAM domains, there is the phosphorylation of CD28 and OX40 for CAR-MNZ and of 4-1BB for CAR-BG2. These different signaling modules activate overlapping but not identical intracellular pathways and different promoters, inducing the expression of specific genes [38,39]. Signaling domain comparisons performed by other groups have suggested that 4-1BB CARs compared to CD28 CARs are less active in vitro but more persistent in vivo in the long term [55]. Nevertheless, different studies have suggested that the advantage given by a specific co-stimulus is strictly dependent on the target antigen [56,57].

The temporal constraints within the mouse model limited our capacity to observe the potential prolonged persistence of CARCIK-BG2 compared to CARCIK-MNZ over time. Moreover, the use of CAR-modified CIK cells, rather than standard T-cells, constitute a new platform that may overcome some of the complications, in particular GvHD or insufficient tumor infiltration, posed by CAR-T cells, although a formal proof of these advantages in humans is still lacking. The ability to use an allogenic source for CIK or CARCIK production is an important point of strength, since CAR-T cells from heavily pre-treated patients may fail to expand or be exhausted [58]. Allogenic CARCIK-CD19 cells have demonstrated to be safe in the phase I/IIa clinical trial, in which no GvHD has been reported and only mild toxicities have been described, confirming the CIK cells safety profile [26]. The safety and efficacy data of this first protocol have led to the design and approval of a second protocol which foresees the repeated infusion (up to two) of CARCIK-CD19 cells (EudraCT number 2020-005025-85). This clinical trial is ongoing.

We acknowledge that our CAR constructs carried multiple differences, including different promoters, signaling modules, and spacers. This study therefore has limitations regarding the comparison of the two CAR-CD19 molecules. Further work will be required to analyze each of these aspects individually.

Overall, the functional comparison and comprehensive analysis provided in this study demonstrate the effectiveness of all three strategies conceived to enhance CIK cell efficacy against a CD19^+^ target. These findings offer valuable insights into the potential for refining CIKs effector functions by manipulating CAR variables or utilizing BiTEs.

## Data Availability

Data are available on request from the corresponding author.

## Supplementary Materials

The following supporting information can be downloaded at: https://www.mdpi.com/article/10.3390/antib13030071/s1. Figure S1: In vitro functional activity against Daudi cell line; Video S1: Immunological Synapse, CIK and REH cells; Video S2: Immunological Synapse, CIK and REH cells in presence of blinatumomab; Video S3: Immunological Synapse, CARCIK-MNZ and REH cells; Video S4: Immunological Synapse, CARCIK-BG2 and REH cells.
