# Supplementary material for: Functional Activity of Cytokine-Induced Killer Cells Enhanced by CAR-CD19 Modification or by Soluble Bispecific Antibody Blinatumomab
Source: Antibodies (Basel). 2024 Aug 30;13(3):71. doi: 10.3390/antib13030071 (PMC11417890; doi:10.3390/antib13030071)
Supplement: Supplementary file 1 [file antibodies-13-00071-s001.zip › Zaninelli et al_Supplementary_Rev.pdf]

Functional activity of CIK cells enhanced by CAR-CD19 modification or by soluble bispecific antibody blinatumomab

Running title: CIK cells enhanced with CAR-CD19 or blinatumomab

Silvia Zaninelli[1], Silvia Panna[1], Sarah Tettamanti[2], Giusi Melita[2], Andrea Doni[3], Francesca D'Autilia[3], Rut Valgardsdottir[1], Elisa Gotti[1], Alessandro Rambaldi[4,5], Josée Golay[1*] and Martino Introna[1].

[1] Center of Cellular Therapy "G. Lanzani", Division of Hematology, ASST Papa Giovanni XXIII, Bergamo, Italy

[2] M. Tettamanti Center, Fondazione IRCCS San Gerardo dei Tintori, Monza, Italy

[3] Unit of Multiscale and Nanostructural Imaging, IRCCS Humanitas Research Hospital, Rozzano, Milano, Italy

[4] Hematology and Bone Marrow Transplant Unit, ASST Papa Giovanni XXIII hospital, Bergamo, Italy

[5] Department of Oncology and Hematology, Università degli Studi di Milano, Milan, Italy

[*]**Corresponding author**: Josée Golay
Center of Cellular Therapy "G. Lanzani", Presidio M. Rota, Via Garibaldi 11-13, 24128 Bergamo, Italy.
E-mail: jgolay59@gmail.com
Tel: +39 035-2278637
Fax: +39 035-2278674

Supplementary Materials

Imaging of immunological Synapse

CIK and CARCIK-CD19 effectors and CD19$^+$ REH target cells were labelled with CellTracker™ Green CMFDA Dye and CellTracker™ Red CMTPX Dye (ThermoFisher), respectively. Effector and target cells were co-cultured in triplicate at a 3:1 E:T ratio in a poly-lysine coated 24-well glass (cover thickness of 0.17±0.005mm bottom) plate (Cellvis-In Vitro Scientific; USA). Images were acquired using a DMI8 Leica microscopy system equipped with a 20X objective (0.40NA HC PL FLUOTAR L). For each time frame, three consecutive but non-overlapping images for each channel and condition were acquired after sequential illumination with the Lumen 200 Fluorescence System (Prior Scientific Inc., Rockland, MA, USA) and collection of the signal contribution for Green CMFDA Dye (Em. 495/517 nm) and Red CMTPX Dye (Em. 550/580 nm) using an ORCA-Flash 4.0 V3 Digital CMOS camera (C13440-20CU; Hamamatsu, Milan, Italy). Images were acquired every 6.5 min up to 1.5 h using the Leica Application Suite X software (LASX; v 3.5.5.19976). All the experiments were performed under climate control (37 °C, 5% CO_2 in a humidified atmosphere; Okolab, Italy). Images were imported into Imaris software (version X64, 9.7.2; Bitplane) and CIK and CARCIK-CD19 cells were reconstructed as isosurface creation, on the basis of thresholded voxels and each cell associated its ID. The CD19$^+$ REH target cells were reconstructed using the spot detection module on the basis of a single cell diameter of 12-14μm and its ID applied to each. A maximum gap size of 3μm was applied, in order to follow each cell in different time frames. Spots of CIK and CARCIK-CD19 cells were automatically quantified as interacting cells with CD19$^+$ REH surfaces within a distance of 0-7μm, which identified contacting cells, to non-interacting cells with a distance to CD19$^+$ REH surfaces of more than 7μm. The results are expression of the accumulation of the frequency of synaptic events of CIK and CARCIK-CD19 on total effector cells.

Supplementary Figure

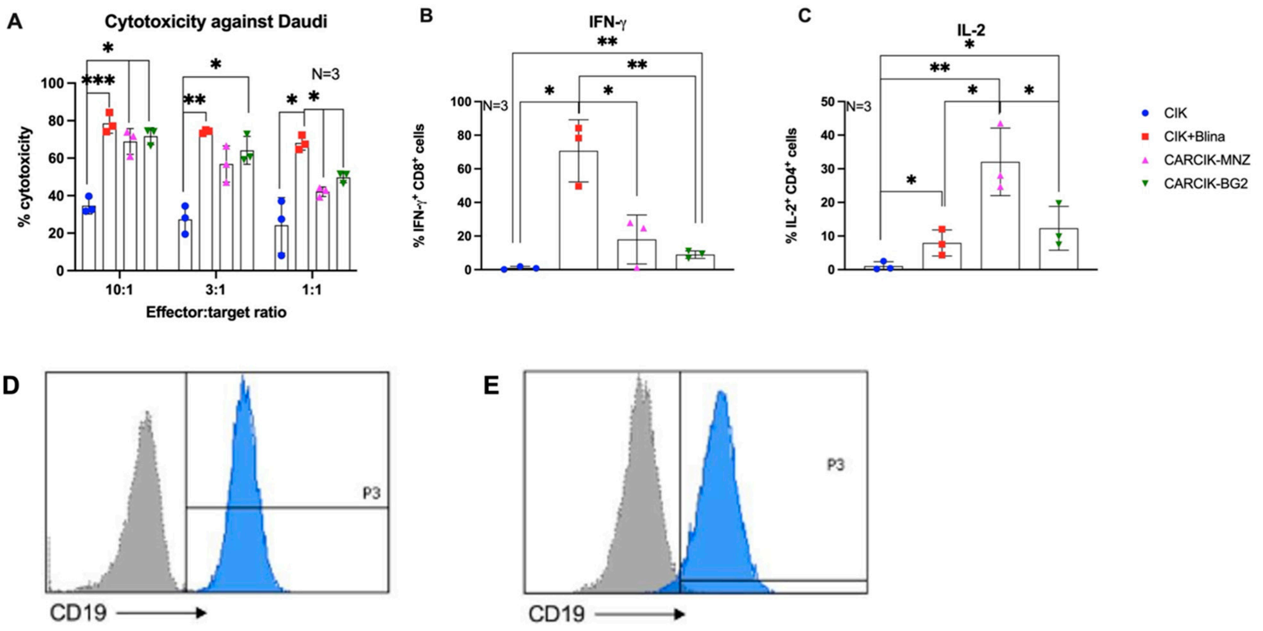

Supplementary Figure S1 - *In vitro* functional activity against Daudi cell line. (A) Comparison of the killing activity in vitro using Calcein release assay. CIK (blue bars), CIK in presence of 10 ng/ml blinatumomab (red bars), CARCIK-MNZ (pink bars) and CARCIK-BG2 (green bars) at the end of culture were used as cytotoxic effector cells against the Daudi cell line at a 10:1, 3:1 and 1:1 E:T ratio. Percentage target lysis is shown as mean and standard deviation of three experiments. (B-C) IFN-γ and IL-2 production was determined by intracytoplasmic flow cytometry after 6 hours co-culture at 1:1 E:T ratio with Daudi cell line. Percentages of positive cells are shown as mean and standard deviation of three experiments. Columns represent the mean, bars the standard deviation. (D) Flow cytometry histograms of CD19 expression on REH cell line in blue and the isotype control in grey (99,6% CD19+, MFI 3356). (E) Flow cytometry histograms of CD19 expression on Daudi cell line in blue and the isotype control in grey (96% CD19+, MFI 3301). (*$p<0.05$, **$p<0.01$, ***$p<0.001$).

Supplementary Videos

Video S1: Immunological Synapse, CIK and REH cells.

Video S2: Immunological Synapse, CIK and REH cells in presence of blinatumomab.

Video S3: Immunological Synapse, CARCIK-MNZ and REH cells

Video S4: Immunological Synapse, CARCIK-BG2 and REH cells
